# Explore the correlation between cerebral vessel characteristics with cognitive impairment among elder individuals: a community study from China

**DOI:** 10.1186/s12883-021-02492-2

**Published:** 2021-12-11

**Authors:** Wei-Wen Wu, Yang Wang, Jun Xu, Li-Xia Lu, Lin Chen, Gang Wu, Hui Yu

**Affiliations:** 1 Department of Neurology, Qingpu Branch of Zhongshan Hospital, Fudan University, 1158 Park East Road, Qingpu Town, Qingpu District Shanghai, 201700 China; 2 Department of Radiology, Qingpu Branch of Zhongshan Hospital, Fudan University, 1158 Park East Road, Qingpu Town, Qingpu District, Shanghai, 201700 China

**Keywords:** Vascular dementia, Cerebral MRI, CSVD score, Left carotid artery peak systolic velocity (PSV), MMSE

## Abstract

**Background:**

Brain Magnetic Resonance Imaging (MRI) examination of cerebral small vessel disease (CSVD) may help screen vascular cognitive impairment. A recently estimated CSVD score system was suggested to capture the overall CSVD burden. The study aimed to detect the association between systemic evaluation score of cerebral vascular imaging parameters with cognitive functions.

**Methods:**

This was a cross-sectional study in community settings. From October 2017 to September 2018, elder (≧60) residents were recruited through on-site visit in 6 communities from Shanghai, China. The participants underwent brain MRI, carotid ultrasound, laboratory tests of blood and urine samples. Cognitive function was evaluated using Mini-Mental State Examination (MMSE). MRI score of CSVD was calculated according to the 2012 standard for the evaluation of statistical changes in imaging.

**Results:**

Total 171 subjects completed survey and examinations. There were 55 participants diagnosed with cognitive impairment, with a total percentage of 32.2%. Participants with and without cognitive impairment showed significant differences in age, BMI and education level. Cognitive impaired participant had more disease history/comorbidity of hypertension and chronic renal insufficiency, higher level of creatinine, as well as lower level of full blood count (FBC) and alanine aminotransferase (ALT). A significant difference was detected in CSVD score between participants with and without cognitive impairment. Results of linear regression analysis showed significant negative correlations between MMSE score and both left and right carotid artery peak systolic velocity (PSV), however the CSVD score was only borderline (*P* = 0.0566) positively correlated with MMSE. Multivariate linear correlation analysis including all collected risk factor data showed that left carotid artery PSV score was among the independent negative correlated factors of MMSE. Multivariate binary logistic analysis showed that age, education and history of hypertension were the only statistically associated factors of cognitive impairment.

**Conclusions:**

The current study identified high prevalence of cognitive impairment in a Chinese community. In addition, correlations between cerebral vascular disease imaging status and cognitive functions were confirmed although the sample size limited the possibility of screening cognitive impairment with imaging technique.

## Background

Dementia is progressive disease in mental health that may lead to compromised capability of independent living. The population affected by dementia was estimated to be 50 million worldwide in 2018 and the number was anticipated to be tripled by 2050 [1]. The disease causes health concerns as well as great economic burden in society. Aging was the most important risk factor of dementia and senile cognitive impairment has emerged as one of the major public health challenges [2]. Mild cognitive impairment (MCI) represents an intermediate state between normal cognition and dementia, and could be a precursor to dementia [3]. Once diagnosed with MCI, individuals are subjected to markedly higher risk for worsening in several years [4]. Medical attention on persons with cognitive impairment and dementia are critical due to their significant care needs. It is anticipated that the cost due to dementia will approach $4 trillion [5].

Vascular etiology, behind Alzheimer’s disease (AD), is the second most common cause of dementia associated with age, and probably the predominant cause of cognitive impairment in East Asia [6, 7]. Vascular disorder, especially those affect small cerebral vessels, may also participate in cognitive dysfunction due to other pathologies, including AD. Factors correlated with cerebrovascular and neurological impairment, including history or morbidity of cerebrovascular and metabolic disease, lifestyle, as well as genetic variants, may affect the risk of vascular dementia (VaD) [8]. The mechanisms might be related either to subcortical vascular lesions or to the lack of compensatory functional cortical changes [9]. The term vascular cognitive impairment (VCI) was proposed to recognize the cognitive alterations caused by vascular factors, while vascular dementia (VaD) refers to the most severe form of VCI [10]. According to Vascular Impairment of Cognition Classification Consensus Study (VICCCS) guideline [11], major VCI (or VaD) is diagnosed with clinically significant deficits in at least 1 cognitive domain that cause a severe disruption of activities of daily living. While mild VCI refers to impairment in at least one cognitive domain but mild to no impairment in activities of daily living [11]. This new definition aligns with revised terminology in DSM-V, which distinguishes between major and minor neurocognitive disorders [8]. It has been reported that depression appeared to be common prior to the onset of vascular dementia due to mood effects caused by ischemic damage on frontal cortical-subcortical circuits, recognized as vascular depression (VD). VD is closely related to cognitive impairment and cerebrovascular pathology. It might have neurophysiological impact on deterioration of cognitive functions, as well as on specific cortical circuits in subcortical vascular disease. For example, white matter lesions (WMLs) are more common and severe in patients with depression [12].

Detailed clinical diagnosis of cognitive impairment is a complex process involving multidimensional assessment of neurophysiological parameters and mental abilities [3]. In practical situations, cognitive testing is more often accomplished using validated screening instrument, such as MMSE [13] or MoCA [14]. Still, the screening and testing of cognitive impairment in practical clinical conditions are insufficient therefore require more auxiliary methods.

Cerebral imaging technique such as brain Magnetic Resonance Imaging (MRI) is required for clinical diagnosis of vascular cognitive impairment (VCI) [15]. Previous studies have indicated the correlations between cerebral vascular imaging appearance with cognitive functions [16, 17]. It has been suggested that vascular brain injury (VBI) imaging indications, such as white matter hyperintensities (WMH) and perivascular spaces (PVS) burdens were associated with higher risk of dementia [18]. The leukoaraiosis and disability (LADIS) study is a European multicenter collaboration started in 2001 aiming at assessing the value of white matter changes (WMC) in predicting disability in elders. The main results of the LADIS study have demonstrated that severe WMC predicted decline of cognitive and motor performances, depressive symptoms associated with aging and cerebrovascular diseases [19, 20].. Markers of cerebral SVD can be individually assessed on brain MRI imaging. However, a total SVD burden score might capture the overall effect of SVD on the brain better than by considering individual features separately. In a most recent analysis of LADIS cohort, it was suggested that a combined measure of global burden of small vessel disease-related brain changes, which extracted with automatic imaging segmentation, was associated with VCI [21]. Yet, a practical comprehensive evaluation system for cerebrovascular imaging is still lacking.

Recently, a pragmatic estimate of total cerebral SVD burden were developed by summing the presence of silent SVD MRI markers including asymptomatic lacunar infarcts, white matter lesions, cerebral microbleeds, and enlarged perivascular spaces, yielding a 0 to 4 “total SVD score” [22]. The scoring system (CSVD score) captures the overall effect of CSVD on the brain, rather than estimating only one or two individual CSVDs separately [23]. This total SVD score was associated with blood pressure and cognition [24], as well as linked with risk factors for CSVD and with cognitive dysfunction in lacunar stroke patients, and it might be helpful for the rapid quantification or stratification of CSVD [22]. It has been shown that imaging changes in cerebral small vessel disease under the cortex are also associated with increased incidence of AD [25]. And the correlation between CSVD and cognitive impairment in stroke patients have also been reported [26]. However, analysis of the correlation between these imaging features and cognitive functions in general elder populations are still few.

In current study, based on a hypothesis that cerebral vascular characteristics are correlated with cognitive impairment, we performed a community based study, aiming to explore the correlation between cerebral vascular imaging features (including lacunes, new small subcortical infarct, enlarged perivascular spaces (EPVS), severity of white matter hyperintensities (WMH), cerebral microbleeds (CMBs), and total MRI score of CSVD) with cognitive functions in elder Chinese population.

## Method

### Study design and subjects

This was a cross-sectional study in community setting aiming to assess the prevalence, risk factors and correlation with cerebral small vessel disease appearance of cognitive impairment in elder residents. From October 2017 to September 2018, elder residents were recruited through on-site visit of 6 communities in Shanghai, China, including:

- Jinze Community, No. 271, Nurture Road, Jinze Town, Qingpu District, Shanghai, China;
- Xujing Community: No. 1088, Xumin Road, Xujing Town, Qingpu District, Shanghai, China
- Xianghuaqiao Community: No.1, Lane 1195, Xinqiao Road, Qingpu District, Shanghai, China
- Daying Community, Xianghuaqiao Street, Qingpu District, Shanghai, China;
- Huaxin Community: 800 Huazhi Road, Huaxin Town, Qingpu District,Shanghai, China;
- Liantang Community: No. 3619, Zhufeng Road, Liantang Town, Qingpu District, Shanghai, China;
- Shenxiang Community: No 39 Quchi Road, Zhujiajiao Town, Qingpu District, Shanghai, China;

Each community were with a population from 40,000 to 200,000. Briefly, flyers and other forms of notices were distributed with the help of neighborhood committees in each community in a period of 2 weeks before an on-site public free health consulting for elder people was given in each community. The attendants of all the event were registered and then 300 participants within the stage age requirement without reported significant diseases were randomly selected and further recruited by a door-to-door visit.

The participants were considered eligible if they have a permanent resident status in Qingpu district, age ≥ 60 years, volunteered to join the study and sign the informed consent. The excluded individuals were those with a history of stroke or diagnosed dementia; history of brain-related diseases such as brain tumors and hydrocephalus; small blood vessel related white matter lesions such as multiple sclerosis; MR contraindications such as cardiac implant pacemakers, implantable defibrillators, cardiac stents, cochlear and other metal implants, claustrophobia; those who were unable to cooperate independently to complete the study; and other serious physical and mental illnesses that may lead to loss of follow-up.

The participants underwent examinations including brain MRI, laboratory tests of blood and urine samples, as well as questionnaire surveys of cognitive functions. All participant signed informed consent form and the study was approved by the Ethical Committee of Zhongshan Hospital affiliated to Fudan University, Qingpu Branch (2017–30).

### Subjects assessment

Cognitive function was evaluated with Mini-Mental State Examination (MMSE) [27]. The validity and reliability of Chinese version of MMSE has been verified previously [28]. The MMSE scale is composed of a total of 30 items, including instant memory, delayed recall, attention and computational power, orientation, object naming, reading comprehension, speech comprehension, language retelling, speech expression, and visual space ability, with each item score 1 point, and 1 extra point added if the patient’s education years are ≤12 years. Cognitive impairment was categorized as following: 25–30, none; 20–25, mild; 10–20; moderate; 0–10, severe. A cutoff of ≤24 points was used as recognition of cognitive impairment [27, 29].

### Brain MRI assessment of small vessel disease status

All patients underwent routine T1 weighted image (T1WI), T2 weighted image (T2WI), Diffusion-weighted imaging (DWI), Fluid-attenuated inversion recovery imaging (FLAIR), and Susceptibility weighted imaging (SWI) examinations using the Umr770 (3.0 T) MRI. The SWI parameter settings were: deflection angle 15°; repetition time 30 ms; echo time 20 ms; 230 mm × 230 mm field of view; matrix 448 × 380; 1 mm layer with 1 mm layer spacing.

All imaging changes related to CSVD (cerebral small vessel disease) were defined according to the 2012 standard for the evaluation of statistical changes in imaging [30]:

- Lacunes: Cystic lesions with a diameter of 3–15 mm, with low signal on T1WI and FLAIR images, and high signal on T2WI images.
- New small subcortical infarct: A high-signal lesion with a diameter of 3–20 mm on DWI.
- Enlarged Perivascular Spaces (EPVS): cystic lesions <3 mm in diameter, with low signal on T1-weighted images and FLAIR images, and high signal on T2-weighted images.
- Severity of white matter hyperintensities (WMH) was assessed using the Fazekas scale [31]: 1) Score of ventricular high signal score: 0 point: no lesions; 1 points: cap-like or pencil-like thin layer lesions; 2 points: the lesion shaped as a smooth halo; 3 points: irregular high signal around the ventricle, extending to the deep white matter. 2) Deep white matter signal: 0 points: no lesions; 1 points: point lesions; 2 points: lesions begin to fuse; 3 points: large area fusion of lesions.
- Cerebral microbleeds (CMBs): a. GRE-T2*WI or SWI sequence with low signal loss; b. Round or oval; c. Clear border; d. Small volume (2 to 5 mm) Or 2 to 10 mm); e. At least 1/2 of the lesion is surrounded by the brain parenchyma.
- MRI score of CSVD [22]: 1) WML: Fazekas scale ≥2 points, add 1 point; 2) sulcus ≥1, add 1 point; 3) deep CMBs ≥1, add 1 point; 4) basal ganglia EPVS >10, add 1 points; total score 0–4.

### Clinical and laboratory variables

We collected the participant’s data including basic demographic information, disease history, family history, smoking and drinking status. The medical conditions of participants including current comorbidity and medications were also collected. In addition, participants were asked to voluntarily take physical examinations and laboratory tests of blood and urine samples to assess traditional risk factors of cognitive impairment/cerebral vascular diseases.

Carotid ultrasonography was performed on each participant to measure the carotid artery intima-media thickness, as previously described [32].

### Statistical analyses

Statistical analyses were performed using the Windows SPSS software package (version 20.0, IBM Corporation, Armonk, NY, USA). The differences between baseline characteristics and cerebral vessel characteristic parameters were compared between patients with and without cognitive impairment, with the Mann-Whitney U test used to compare continuous variables, and Fisher’s exact test or the chi-square test used to compare categorical variables, as appropriate. As exploratory analysis for penitential correlated factors of cognitive functions, multivariate linear correlation test was performed to detect association between baseline and cerebral vascular characteristics with MMSE score. To determine the risk factors of cognitive impairment, multivariate binary logistic regression test was applied to identify independent correlating factors for cognitive impairment. A probability value of *p* < 0.05 was considered statistically significant.

## Results

### Participants

After screening with eligible criteria, total 171 subjects completed survey and examinations. There were 48 participants diagnosed with mild, 7 with moderate cognitive impairment, with a total percentage of 32.2%.

As shown in Table 1, participants with cognitive impairment showed significant differences in age, BMI, education level and labor status. Cognitive impaired participant were also identified with higher level of HDL and UCR, and lower levels of full blood count (FBC) and AST/ALT. These results indicated that the cognitive impairment may be associated with other comorbidities or health conditions.

**Table 1:** Demographic and clinical data of participants with different level of cognitive impairment

Characteristics		No (*n* = 116)	Yes (*n* = 55)	*P* value
	Age (years); Mean ± SD	67.1 ± 4.7	69.3 ± 5.1	0.006
Demographic	Gender (n)			0.168
	Male	53	19	
	Female	63	36	
	BMI; Mean ± SD	23.7 ± 2.8	22.1 ± 2.2	0.001
	Marriage status; n			0.034
	Married	109	45	
	Divorced	2	2	
	Widowed	5	8	
	Living status; n			0.215
	Alone	5	2	
	With spouse	14	12	
	With children	70	34	
	With both	27	7	
	Education level; n			0.015
	College	2	0	
	High school	11	2	
	Middle school	34	8	
	Elementary school	51	26	
	None	18	19	
	Income (Yuan/per month); n			0.586
	<1000	2	3	
	1000 ~ 2999	74	31	
	3000–4999	29	16	
	>5000	10	5	
	5	1	0	
	Labor status in work; n			0.040
	Mild	52	14	
	Moderate	60	38	
	Heavy	3	3	
	Smoke status; n			0.224
	None	71	42	
	Mild	1	1	
	Moderate	24	6	
	Heavy	19	6	
	Drinking			0.212
	None	79	43	
	Mild	17	9	
	Moderate	15	2	
	Heavy	5	1	
Medical conditions	Hypertension; n			0.782
	Missing	2	1	
	No	36	20	
	Yes	78	34	
	Hyperlipidemia; n			0.367
	Missing	1	1	
	No	89	48	
	Yes	20	6	
	Unknown	3	0	
	Diabetes; n			0.225
	Missing	0	1	
	No	80	41	
	Yes	36	13	
	TIA; n			0.258
	Missing	1	2	
	No	109	53	
	Carotid stenosis; n			0.219
	Missing	1	2	
	No	110	53	
	Yes	3	0	
	Brain trauma; n			0.425
	Missing	1	2	
	No	110	52	
	Yes	3	1	
	PAD; n			0.682
	Missing	1	1	
	No	112	53	
	Yes	1	1	
	CVD; n			0.466
	Missing	1	2	
	No	107	52	
	Yes	2	1	
	AF; n			0.346
	Missing	1	2	
	No	114	53	
	Yes	1	0	
	Chronic renal insufficiency; n			0.148
	Missing	1	2	
	No	115	52	
	Yes	0	1	
	Gout/high uric acid; n			0.434
	Missing	1	2	
	No	113	52	
	Yes	2	1	
	Tumor; n			0.217
	Missing	1	2	
	No	112	53	
	Yes	3	0	
Physical conditions and laboratory examinations	HR; Mean ± SD	81.3 ± 77.2	78.8 ± 9.7	0.812
	SBP; Mean ± SD	128.8 ± 12.0	128.6 ± 10.1	0.895
	DBP; Mean ± SD	77.8 ± 7.6	77.3 ± 8.5	0.683
	Hcy; Mean ± SD	16.2 ± 5.0	15.3 ± 5.1	0.274
	Cholesterol; Mean ± SD	5.2 ± 0.89	5.2 ± 0.61	0.901
	Triglyceride; Mean ± SD	1.7 ± 0.91	1.8 ± 0.74	0.696
	HDL; Mean ± SD	1.4 ± 0.28	1.5 ± 0.24	0.021
	LDL; Mean ± SD	3.2 ± 0.84	3.1 ± 0.57	0.844
	AST; Mean ± SD	28.8 ± 10.6	24.8 ± 9.1	0.016
	ALT; Mean ± SD	31.0 ± 9.2	25.2 ± 10.7	< 0.0001
	Cystatin C; Mean ± SD	0.98 ± 0.57	0.86 ± 0.18	0.135
	Creatinine; Mean ± SD	64.1 ± 14.7	65.5 ± 15.3	0.586
	Uric acid; Mean ± SD	336.2 ± 67.9	346.9 ± 60.3	0.323
	FBC; Mean ± SD	6.3 ± 1.7	5.5 ± 1.1	0.001
	HbA1c; Mean ± SD	6.0 ± 1.2	6.3 ± 6.9	0.661
	CRP; Mean ± SD	1.8 ± 2.6	1.8 ± 0.98	0.902
	UCR; Mean ± SD	11.1 ± 6.3	15.2 ± 8.7	0.001
	MA; Mean ± SD	33.1 ± 40.7	26.7 ± 26.1	0.290
	UACR; Mean ± SD	22.8 ± 24.1	25.9 ± 22.0	0.429

*TIA* Transient ischemic attack, *PAD* Peripheral artery disease, *CHD* Coronary heart disease, *AF* Atrial fibrillation, *HR* Heart rate, *SBP* Systolic Blood Pressure, *DBP* Diastolic blood pressure, *Hcy* Homocysteine, *HDL* High-density lipoprotein, *LDL* Low-density lipoprotein, *AST* Aspartate aminotransferase, *ALT* Alanine aminotransferase, *FBC* Full blood count, *HbA1c* Hemoglobin A1c, *CRP* C-reactive protein, *UCR* Urine Creatinine, *MA* Microalbumin, *UCAR* Urine albumin-to-creatinine ratio

The cerebral vascular imaging parameters were also compared between participants with and without cognitive impairments. A significant difference was detected in CSVD score (Table 2).

**Table 2:** Comparison between cerebral vascular imaging parameters between participants with different cognitive impairment

Parameters; Mean ± SD	Cognitive impairment		*P* value
	No (*n* = 116)	Yes (*n* = 55)	
LCCA-IMT	0.752 ± 0.092	0.745 ± 0.088	0.6471
LCCA-PSV	6.27 ± 2.51	6.02 ± 1.25	0.4043
RCCA-IMT	0.741 ± 0.088	0.738 ± 0.085	0.8492
RCCA-PSV	6.15 ± 2.71	6.49 ± 6.27	0.6985
LCA-psv	5.12 ± 3.23	5.34 ± 1.36	0.5318
RCA-psv	5.64 ± 5.32	6.64 ± 7.41	0.3704
CSVD	1.59 ± 1.47	1.13 ± 1.38	0.0471

*LCCA-IMT* Left common carotid artery intima-media thickness, *LCCA-PSV* Left common carotid artery peak systolic velocity, *RCCA-IMT* Right common carotid artery intima-media thickness, *RCCA-PSV* Right common carotid artery peak systolic velocity, *LCA-psv* Left carotid artery peak systolic velocity, *RCA-psv* Right carotid artery peak systolic velocity, *CSVD* Cerebral small vessel disease score

Linear correlations with MMSE cognitive function score have been often utilized to explore potential risk factors of cognitive impairment [33, 34]. To further explore the association of cognitive function with cerebral vascular imaging parameters, linear regression analysis was performed between MMSE score and imaging parameters. As shown in Table 3, significant correlations were detected between MMSE score and both left and right carotid artery peak systolic velocity (PSV) score. However, the CSVD score was only borderline (*P* = 0.0566) correlated with MMSE. Multivariate correlation analysis including all collected risk factor data (Table 4) showed that left carotid artery PSV score was among the independent correlated factors of MMSE. In addition, MMSE was significantly associated with age, gender, marital status, education, income, smoking status, history and medication of hypertension and hyperlipidemia, history of peripheral artery disease (PAD) and coronary heart disease (CHD).

**Table 3:** Univariate linear correlation test between MMSE score and cerebral vascular disease status

		mmse	LCCA-IMT	LCCA-PSV	RCCA-IMT	RCCA-PSV	LCA-psv	RCA-psv	CSVD
mmse	Coefficient	1.0000	0.0014	0.0068	−0.0190	0.0165	−0.1700	−0.1820	0.1465
	*P* value		0.9860	0.9298	0.8055	0.8313	0.0264	0.0172	0.0566
LCCA-IMT	Coefficient	0.0014		0.0083	0.7670	−0.0250	−0.0475	−0.0282	0.1061
	*P* value	0.9860		0.9144	< 0.0001	0.7453	0.5370	0.7141	0.1672
LCCA-PSV	Coefficient	0.0068	0.0083		−0.0678	0.6220	0.3610	0.3180	0.1199
	*P* value	0.9298	0.9144		0.3783	< 0.0001	< 0.0001	< 0.0001	0.1184
RCCA-IMT	Coefficient	−0.0190	0.7670	−0.0678		−0.0381	−0.0370	−0.0462	0.0700
	*P* value	0.8055	< 0.0001	0.3783		0.6206	0.6313	0.5489	0.3630
RCCA-PSV	Coefficient	0.0165	−0.0250	0.6220	−0.0381		0.3190	0.3850	0.0577
	*P* value	0.8313	0.7453	< 0.0001	0.6206		< 0.0001	< 0.0001	0.4539
LCA-psv	Coefficient	−0.1700	−0.0475	0.3610	−0.0370	0.3190		0.0000	−0.0780
	*P* value	0.0264	0.5370	< 0.0001	0.6313	< 0.0001		< 0.0001	0.3108
RCA-psv	Coefficient	−0.1820	−0.0282	0.3180	−0.0462	0.3850	0.7060		−0.0802
	*P* value	0.0172	0.7141	< 0.0001	0.5489	< 0.0001	< 0.0001		0.2970
CSVD	Coefficient	0.1465	0.1061	0.1199	0.0700	0.0577	−0.0780	−0.0802	
	*P* value	0.0566	0.1672	0.1184	0.3630	0.4539	0.3108	0.2970

**Table 4:** Independent correlated factors of MMSE identified with multivariate linear regression analysis

	B	t	*P* value
Gender	2.738973	2.405738	0.0186
Age	−0.19572	−3.40499	0.0011
Marriage status	−1.39785	−3.11385	0.0026
Education level	−2.18476	−6.35842	<0.0001
Income	−1.50688	−3.49387	0.0008
Smoke status	1.539501	3.505515	0.0008
Antihypertensive drugs	0.070463	4.749113	<0.0001
Antilipid oxidant	−6.53721	−2.60101	0.0112
Hypertension	−5.21405	−2.03592	0.0453
Length of hypertension	−0.15209	−3.66463	0.0005
Hyperlipidemia	0.517124	2.305949	0.0239
Peripheral vascular disease	7.042743	2.05852	0.0430
Coronary heart disease	−5.06527	−2.00128	0.0490
LCA-psv	−0.63425	−3.23109	0.0018

Although significant linear correlations with MMSE score has been identified in several factors, when participants were categorized as with or without cognitive impairment according to MMSE score, multivariate binary logistic analysis showed that age, education and history of hypertension were the only statistically independent associated factors of cognitive impairment (Table 5).

**Table 5:** Independent correlated factors of cognitive impairment identified with multivariate logistic regression analysis

	*P* value	Exp(B)	95% CI
Age	0.0022	1.2209	(1.0742, 1.3876)
Education	0.0002	4.3309	(1.9878, 9.4359)
Hypertension	0.0813	1.8195	(0.9282, 3.5664)
Length of hypertension	0.0387	1.1011	(1.0050, 1.2064)

## Discussion

The results in this study showed that there was a significant prevalence of cognitive impairment in the elder population of communities in Qingpu District, Shanghai, China. Age, education, vascular and metabolic disease histories were the common risk for cognitive impairment. Correlations between cerebral vascular imaging parameters, including left and right carotid artery PSV (perivascular spaces) score, and CSVD with cognitive functions have been detected in various settings, with the strongest association found between left carotid artery PSV and MMSE. However, the CSVD score was not statistically significant in recognition of cognitive impairment.

Cognitive impairment is concerned in certain clinical settings such as post-stroke and atrial fibrillation (AF) patients [35]. In addition, it is a great risk factor of elder abuse [36]. This study was aiming mainly to evaluate associated between CVSD imaging characteristics with cognitive impairment, instead of a pure epidemiology estimation of VCI. The study was performed in Shanghai due to cooperation with community enable a relative large regional population based study to be carried. This region in general may represent a population of Southeastern China population with relative better economic and educational levels. Although many studies have been reported on the prevalence of dementia in China, very few was on mild cognitive impairment. Most of these studies have been performed in isolated regions therefore the results showed a broader range of variations. Meta-analysis of 96 observational studies reported that the overall prevalence of dementia in Chinese people aged 60 years and older was 5·30%, higher in rural populations, probably due to the lower educational level. Age and sex also affect dementia prevalence. The prevalence of dementia is higher in Western China and northern China compared to southern China. The prevalence of mild cognitive impairment was reported to be 12·7% for individuals aged 60 years and older across all of the regions. Vascular-related mild cognitive impairment subtypes were the most common (42%) in individuals aged 65 and older. Lower educational level, women and rural area residences were associated with higher prevalence of cognitive impairment [7, 31, 37, 38].. In this study, we identified more than 30% participants with mild and moderate cognitive impairment. In addition, significant percentage of participants in the current study had vascular or metabolic disease history. These results indicate that most the participant may have certain concerns in health status therefore the estimated prevalence of cognitive impairment might have been magnified.

The scope of the current report was to identify whether the vertebral vessel imaging characteristics was correlated with cognitive impairment in general elder population. The MMSE was selected as easy tool for cognitive function evaluation. The other neurological examination was not assessed in this study. This will certainly limit the detailed understanding about the relation between CSVD signs with different sections of cognitive functions. However, although the MMSE has significant limitations [39], it has been reported in LADIS study related cohort that MMSE scores were the only neuropsycological predictor of cognitive impairment in long term [40], indicating that a correlation with MMSE score may represents a relation with cognitive functions.

The results showed that although individual correlations between MMSE score with left carotid artery (LCA)-psv, right carotid artery (RCA)-psv and CSVD score could be detected in various settings, the associations were not very strong. The only independent correlation between MMSE and these features were with LCA-psv in a linear regression analysis. CSVD score was not significantly associated with cognitive impairment. Since the study contain a relatively small sample size for multivariate analysis, and we did not perform any examination of AD symptom or biomarkers, therefore the negative results might have resulted from the limited statistical power. However, based on the results in univariate analysis, we would expect the association might be detected in a larger sample size and this is warranted in our future study.

Various results from LADIS studies initiated since 2001 have demonstrated the correlations between WMC with decline of cognitive and motor performances, depressive symptoms associated with aging and cerebrovascular diseases [19]., as well as with the presence of urinary disturbances, and various neurological abnormalities [20]. It was found that early-stage white matter lesions predict progressive cognitive decline, indicating the importance of early recognition of small vessel disease and incipient vascular cognitive impairment [41], systematically checking for neurological examination abnormalities in older patients may be cost-effective in identifying high risk population [42]. In a recent reported LADIS related study, a combined measure of WMH, lacunar, gray matter, and hippocampal volumes, extracted by automatice imaging segmentation, was suggested to be able to serve as an imaging marker associated with vascular cognitive impairment [21]. Our results are in general consistent with these findings, and suggested that a systematic evaluation of cerebral VSD imaging markers may be important in screening the population at risk of VSI development.

The risk factors for dementia overlap with those for stroke, indicating shared pathological mechanisms [43]. The risk factors for both vascular and Alzheimer’s dementia include increasing age, female sex as well as hypertension and diabetes [1]. There is also evidence linking cholesterol and obesity with elderly dementia [2, 44]. Smoking is linked to an increased risk of cognitive decline [45]. Cerebrovascular diseases are common risk factors for cognitive impairment. In this study, we observed that age, BMI, education level, disease history/comorbidity of hypertension and chronic renal insufficiency, and higher level of creatinine were among the potential correlated factors of cognitive impairment, which was consistent with previous studies. Multivariate linear analysis also detected the association between cognitive functions with traditional risk factors. However, only age, education and hypertension history were among the factors that may discriminate cognitive impairment. Again, these results might be due to the limited sample size and statistical power.

This study was subject to some limitations. First, as mentioned above, the sample size may have limited the statistical power of the multivariate analysis. In addition, many possible interactions among the risk factors exist, which may also interfere with statistical results. Furthermore, as a survey in community, the selection bias caused by the recruitment process and willingness of participants should always be considered.

## Conclusion

In conclusion, the current study identified high prevalence of cognitive impairment in a Chinese community. In addition, correlations between cerebral vascular disease imaging status and cognitive functions were confirmed although the sample size limited the possibility of screening cognitive impairment with imaging technique.

## Data Availability

All data generated or analysed during this study are included in this published article.

## Abbreviations

MMSE: Mini-Mental State Examination
CSVD: Cerebral small vessel disease
FBC: Full blood count
PSV: Peak systolic velocity
ALT: Alanine aminotransferase
PVD: Peripheral vascular disease
CHD: Coronary heart disease
MCI: Mild cognitive impairment
AD: Alzheimer’s disease
VaD: Vascular dementia
VD: Vascular depression
VCI: Vascular cognitive impairment
VICCCS: Vascular Impairment of Cognition Classification Consensus Study
MRI: Magnetic Resonance Imaging
VBI: Vascular brain injury
WMH: White matter hyperintensities
PVS: Perivascular spaces
LADIS: Leukoaraiosis and disability
WMC: White matter changes
T1WI: T1 weighted image
T2WI: T2 weighted image
SWI: Susceptibility weighted imaging
DWI: Diffusion-weighted imaging
FLAIR: Fluid-attenuated inversion recovery imaging
EPVS: Enlarged Perivascular Spaces
CMBs: Cerebral microbleeds
PAD: Peripheral artery disease
CHD: Coronary heart disease
AF: Atrial fibrillation
LCA: Left carotid artery
RCA: Right carotid artery
